# Granulocytic Sarcoma in a Nonleukemic Patient: Place of Radiotherapy and Systemic Therapies

**DOI:** 10.1155/2011/929161

**Published:** 2011-05-10

**Authors:** C. Chargari, J. Jacob, O. Bauduceau, F. R. Ferrand, T. De Revel, L. Védrine

**Affiliations:** ^1^Unité d'Oncologie Médicale et de Rayonnement, Hôpital d'Instruction des Armées du Val-de-Grâce, Paris 75230, France; ^2^Department d'Hématolgie, Hôpital d'Instruction des Armées Percy, Clamart 92141, France

## Abstract

Granulocytic sarcoma is a rare extramedullary tumour, which most often occurs in the course of an acute or chronic leukaemia or myeloproliferative disorders. Rarely it is found before peripheral blood or bone marrow evidence of leukemia is present. We report an unusual case of acute paraplegia at first presentation of a spinal epidural granulocytic sarcoma without any haematological disorder. Therapeutic strategies are discussed in the light of the literature.

## 1. Introduction

Granulocytic sarcoma is a rare extramedullary tumour composed of immature cells of the granulocytic series [1]. Most cases of granulocytic sarcoma occur in the course of an acute or chronic leukaemia or myeloproliferative disorders. It is rarely found before evidence of leukemia. Spinal cord invasion by granulocytic sarcoma is also uncommon [2]. We report an unusual case of acute paraplegia at first presentation of a spinal epidural granulocytic sarcoma without any haematological disorder and treated with association of neurosurgery and radiation.

## 2. Case Report

A previously healthy 41-year-old man was hospitalized for acute paraplegia. Four weeks before, he had developed a localized back pain and gradual weakness of both legs. Physical examination revealed complete paralysis of left lower extremity, 1/5 paraparesis of right lower extremity, symmetric severe hypoesthesia with supraumbilical sensitive level, reduced deep tendon reflexes in the lower extremities, and loss of sphincter tone. Cutaneous plantar reflexes were both with flexion. Laboratory evaluation revealed a white blood cell count of 12800/mm^3^, a haemoglobin level of 11.6 g/dL, and a platelet count of 327000/mm^3^. 

**Figure 1:**
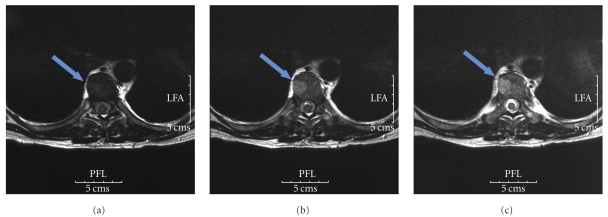
Magnetic resonance imaging of the thoracolumbar spine revealing an anterior and circular epidural mass of T8 (axial view).

**Figure 2:**
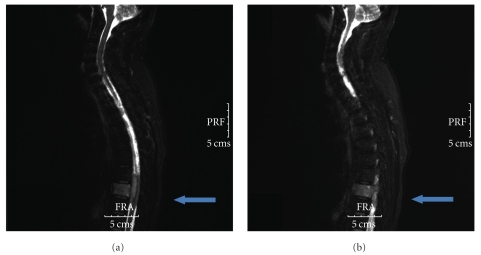
Magnetic resonance imaging of the thoracolumbar spine revealing an anterior and circular epidural mass of T8 (sagittal view).

Magnetic resonance imaging (MRI) of the thoracolumbar spine revealed an anterior and circular epidural mass of T8 with spinal block (Figures 1 and 2). He received high dose of corticosteroids and underwent emergency decompressive laminectomies of T7 and T8.

Anatomopathologic observations revealed a population of immature large cells with ovale and slightly nuclei and eosinophilic cytoplasm. Immunohistochemical staining revealed the strong expression of myeloperoxidase (MPO) in 30% of tumour cells, weaker expression of MPO in the same rate of cells, strong positivity of macrophages for CD68, strong positivity of neutrophil polynuclears for CD15, and positivity of vascular structures for CD34. The tumor cells proliferation was negative for B-lymphoid markers (CD20, CD79a, Mi18) and T markers (CD1a, CD3, CD5). Ki 67 evaluation was of 80% of the tumor cells. Altogether, these phenotypic and anatomic features sustained the diagnosis of granulocytic sarcoma.Two medullograms revealed neither morphologic abnormalities, nor excess of blasts. Bone marrow examination showed slightly granulocytic hyperplasia but neither leukemic infiltration nor evidence of myelodysplasia.

In the early postoperative period, the patient showed fast recovery of paraplegia but persistent partial hypoesthesia. Furthermore, there was in a few days an improvement of sphincter abnormalities and pain disappeared. Spinal radiotherapy (RT) of 40 Gy to the thoracic spine was delivered 15 days after surgery from T5 to T10, given in 16 fractions, with no acute toxicity. 

Unfortunately, after one-year followup, the patient has developed an acute AML-4 (acute myeloid leukaemia type 4) with chromosome 16 inversion. He was referred for high doses of idarubicine and aracytine as first line chemotherapy (CT).

## 3. Discussion

Also known as chloroma by the green colour the myeloperoxidase enzyme gives off, granulocytic sarcoma is a rare tumor that is composed of immature granulocytes in extramedullary sites. It usually presents concomitantly with or in the course of acute myelogenous leukaemia (AML), blastic phase of chronic myelogenous leukaemia, or myelodysplastic syndrome [1, 2]. Granulocytic sarcoma is reported in 0.7–9 per cent of patients with acute or chronic myelogenous leukaemia [3]. Most of them are children or young adults. Their survival is shorter than that of patients without granulocytic sarcoma. Granulocytic sarcoma preceding the evidence of leukaemia is very rare. It is reported to occur in 0.6% of AML [4]. In nearly half of cases without any haematological disorder, acute leukaemia develops in a short delay, since the mean interval between initial diagnosis and the onset of acute leukaemia is about 10 months [2]. Due to rarity and because morphologic evidence of granulocytic differentiation is frequently lacking, about 75% of nonleukemic granulocytic sarcoma are misdiagnosed as other common malignancies, as lymphoma (50%) or nonhematopoietic neoplasm (25%) [5]. It should be noticed that the green colour rapidly fades when exposed to air, which contributes to misdiagnosing granulocytic sarcoma [6].

Granulocytic sarcomas usually occur in the bone, skin, and lymph nodes. While bony involvement is most common in the orbit, sacrum, spine, or ribes, presentation as an epidural granulocytic sarcoma with cord compression in aleukemic patients is extremely rare. We could find only 15 such cases described in the literature. The most frequent initial symptoms of paraspinal granulocytic sarcoma were back pain and radicular pain. Weakness and/or paralysis of one or more extremities were reported in approximately 80%,with the thoracic level being the most commonly affected site.

Nonleukemic granulocytic sarcoma represents a therapeutic dilemma because the optimal therapy is unclear [7]. More than 80% of patients with nonleukemic granulocytic sarcoma who are treated by local therapy alone (either surgical excision or RT or both therapies) will develop overt leukaemia in a few months following local treatment [4, 8]. A retrospective Canadian study of 90 cases of nonleukemic granulocytic sarcoma [9] provided further evidence of systemic therapy early in the course of nonleukemic granulocytic sarcoma, with a decreased probability of developing AML if receiving CT (41% versus 71%, *P* = .001). Moreover, the median time from diagnosis of nonleukemic granulocytic sarcoma to leukaemia was 36 months for patients treated with CT (versus 6 months in untreated patients). Overall survival was twice longer in the patients receiving CT than for patients who did not (median survival of 25 months if treated with CT versus 13 months in patients untreated). This study showed that the local treatment alone had no effect on survival. Another retrospective study of 74 cases [10] showed that the nonleukemic period after the diagnosis of granulocytic sarcoma was 12 months for patients receiving antileukemic induction CT versus 3 and 6 months for patients receiving only surgical resection or RT, respectively. Impact of allogenic or autologous bone marrow transplantation has not been defined.

In case of spinal cord compression in a leukemic patient, treatment of choice is to deliver RT and CT before surgery. However, the optimal management of paraspinal granulocytic sarcoma in nonleukemic patients is more controversial. For nonleukemic patients without radiological or clinical evidence of cord compression, granulocytic sarcoma should be primarily treated with high dose of corticosteroids followed by systemic AML type CT and RT considering the high chemo- and/or radiosensitivity of the tumour [6].

For aleukemic patients with rapidly progressive neurological signs and radiological evidence of cord compression (as in the case we reported here), emergency laminectomy could be logically recommended [11]. Considering the risk of subsequent leukaemia and the poor outcome after only local treatment, early postoperative systemic AML-type CT seems reasonable. Considering the high risk of subsequent development of overt leukaemia, radiation therapy alone does not seem to be enough, and it was suggested that patients with isolated granulocytic sarcoma should be treated, after local treatment, as if they had acute myelogenous leukaemia. However, the benefit of delivering CT (which is a associated with high rate of severe treatment-related toxicities) has not been prospectively demonstrated. Further assessments are therefore encouraged, but large randomized trials will not be possible, due to the disease rarity. In the last guidelines published by the National Comprehensive Cancer Network, it was recommended that for patients presenting with solitary extramedullary disease without overt marrow disease, the initial treatment should still be based on systemic chemotherapy. Radiation therapy or surgical procedure would be incorporated in case of emergency situation [12]. For elderly patients, another strategy could be to treat locally granulocytic sarcoma, then treat systematically when AML develops, typically within one to three years. Anyway, the decision of treating systematically patients before the evidence of AML should be taken after multidisciplinary discussion, since about 20% of nonleukemic patients will remain disease-free after local therapy only. 

## 4. Conclusion

Granulocytic sarcomas are rarely observed before the diagnosis of any hematological malignancy. Frequently misdiagnosed, most of them are harbingers of acute leukaemia developing in next few months. Early diagnosis may allow patients affected to be more efficiently treated and potentially increasing their survival. Presentation as an epidural granulocytic sarcoma and cord compression in leukemic patients is extremely rare and requires emergency laminectomy with postoperative RT when neurological symptoms are present. Although systemic antileukemic CT seems to be a promising strategy, the benefit of such strategy needs to be further assessed.
